# MDPbiome: microbiome engineering through prescriptive perturbations

**DOI:** 10.1093/bioinformatics/bty562

**Published:** 2018-09-08

**Authors:** Beatriz García-Jiménez, Tomás de la Rosa, Mark D Wilkinson

**Affiliations:** 1 Center for Plant Biotechnology and Genomics UPM - INIA, Universidad Politecnica de Madrid, Madrid, Spain; 2 Department of Computer Science, Universidad Carlos III de Madrid, Madrid, Spain

## Abstract

**Motivation:**

Recent microbiome dynamics studies highlight the current inability to predict the effects of external perturbations on complex microbial populations. To do so would be particularly advantageous in fields such as medicine, bioremediation or industrial scenarios.

**Results:**

MDPbiome statistically models longitudinal metagenomics samples undergoing perturbations as a Markov Decision Process (MDP). Given a starting microbial composition, our MDPbiome system suggests the sequence of external perturbation(s) that will engineer that microbiome to a goal state, for example, a healthier or more performant composition. It also estimates intermediate microbiome states along the path, thus making it possible to avoid particularly undesirable/unhealthy states. We demonstrate MDPbiome performance over three real and distinct datasets, proving its flexibility, and the reliability and universality of its output ‘optimal perturbation policy’. For example, an MDP created using a vaginal microbiome time series, with a goal of recovering from bacterial vaginosis, suggested avoidance of perturbations such as lubricants or sex toys; while another MDP provided a quantitative explanation for why salmonella vaccine accelerates gut microbiome maturation in chicks. This novel analytical approach has clear applications in medicine, where it could suggest low-impact clinical interventions that will lead to achievement or maintenance of a healthy microbial population, or alternately, the sequence of interventions necessary to avoid strongly negative microbiome states.

**Availability and implementation:**

Code (https://github.com/beatrizgj/MDPbiome) and result files (https://tomdelarosa.shinyapps.io/MDPbiome/) are available online.

**Supplementary information:**

Supplementary data are available at *Bioinformatics* online.

## 1 Introduction

This manuscript addresses an important challenge in microbiome analysis (Bashan *et al.*, 2016; Bradley and Pollard, 2017; Gilbert *et al.*, 2016): precise description of longitudinal microbiome variability and dynamics. It responds to the call for a ‘microbial Global Positioning System (GPS)’, originally suggested by Gilbert *et al.* (2016), where the start and end states of the microbiome for an individual subject could be defined and located, and the optimal route from start to end clearly mapped. High-throughput sequencing has enabled the study of metagenomics—determination of microbial compositions more precisely and rapidly than bacterial culture techniques. Metagenomics analyses of the same population over time may reveal the detailed dynamics within complex bacterial communities, interactions between microbes and the influence of external perturbations. Inferring microbial dynamics from temporal metagenomics data is, however, a very challenging task (Cao *et al.*, 2017) and there are relatively few studies and little knowledge about microbiome dynamics. Nevertheless, it should be possible to utilize such data to construct models aimed at *in silico* prediction of perturbation-outcomes (Fritz *et al.*, 2013).

It is well-recognized that predictable microbial compositions are associated with important traits such as health (Gilbert *et al.*, 2016; Shankar, 2017), and recent studies have supported alteration of the microbiome as ‘therapy’ (Bashan *et al.*, 2016; Cao *et al.*, 2017). Therefore, an example of where predictive microbiome models would be useful would be a hospital critical-care ward. Patients suffering from sepsis often die before traditional bacterial cultures can be returned, and often the causative agent is never identified. With little information about the cause of the infection, a wide range of high-impact clinical interventions are applied to the patient in the hope that one might prove effective. Unfortunately, the resulting disruption—including to the normal microflora of the patient—often leads to serious complicating illnesses such as pneumonia (Boyd *et al.*, 2014). *In silico* models of the microbiome could, therefore, provide badly needed and rapid feedback on the efficacy of an ongoing treatment regime, or better still, guidance on a specific course of interventions—for example, a personalized and contextually sensitive sequence of drugs and/or food—that could lead the patient safely back to health. Outside of medicine, intensive agriculture has significant negative impacts on the environment through, for example, greenhouse gas emissions and the application of large amounts of pesticides and fertilizer, and the resulting contamination of groundwater (Canter, 2017). The plant-associated microbial populations available to be engineered are the root-associated (endophytic) microbes in the rhizosphere, with the objective of improving plant health or nutrition, improving soil fertility and/or promoting low-impact, sustainable farming.

Current approaches to microbiome engineering might be considered ‘extreme’, for example, faecal microbiome transplantation (FMT). Less extreme, but nevertheless relatively direct, pre- and pro-biotics are used in the farming industry to engineer animal gut microbiomes in order to avoid the pathogen resistance resulting from antibiotic treatments. Acknowledging that microbiome engineering was still in its infancy, Foo *et al.* (2017) proposed to engineer the microbiome through perturbations to recover microbial communities from a state of dysbiosis. Achieving this, however, will require a deeper understanding of, and tooling to measure, microbiome states and responses.

It is known that elements such as natural variations and stress factors modify microbiota composition (Weiss and Hennet, 2017), however it is an open problem to discover which factor(s) lead to which specific composition change, and how and why that happens. The review of Faust *et al.* (2015) lists a series of observations regarding temporal changes in the microbiome that are particularly relevant to the work we report here. First, microbial diversity tends to be stable over time in the same environment. Second, microbial communities evolve through stable states that change due to (i) external perturbations (e.g. diet), (ii) direct modifications (e.g. antibiotics, probiotics) or (iii) transient perturbations (e.g. microbial interactions). Third, subsequent to a perturbation, the community may return to its original state, or may remain in the new (or another) state. Finally, those communities exhibit a priority effect, where the existence of certain strains will prevent specific other taxa from establishing themselves in a community. These observations are encouraging, in that they suggest that it should be possible to build predictive state-change models. Moreover, they reveal that not all state-changes are possible, thus indicating that a desired state-change might require sequential, planned interventions.

There are few large, publicly available longitudinal metagenomics datasets that could be used to design such models. Most datasets span short periods of time (weeks), though some span several years. Most studies are focused on the human gut, but unfortunately, they seldom include longitudinal metadata (i.e. possible perturbations) associated with each sample. Gibbons *et al.* (2017) suggested that current time series metagenomics datasets are not rich enough to be used to explain population dynamics. Gilbert *et al.* (2016) analysed which sampling frequency would be required in a microbiome time series to capture the microbiota dynamics with sufficient detail to be applied in medicine, concluding that it is an open problem. Nevertheless, there have been attempts at modelling microbiome dynamics. Lotka–Volterra (LV) models were the first to predict changes in community composition only based on pairwise microbial interactions (Stein *et al.*, 2013), with some posterior improvements based on generalized LV (Cao *et al.*, 2017). State transition diagrams have also appeared in some temporal dynamics metagenomics studies (Ding and Schloss, 2014; Gajer *et al.*, 2012). These, however, do not specify the role of distinct perturbations.

Tools that could be applied to investigating microbiomes are usually not suited to longitudinal datasets; other tools are specialized for generic time series, not taking biological peculiarities into account thus limiting their applicability to metagenomics data; and finally others are focused on microarray analyses, which is a distinct problem from that of metagenomics [see descriptions in (Baksi *et al.*, 2018)]. MDSINE (Bucci *et al.*, 2016) is the tool most-adapted for time-series microbiome data. MDSINE includes time-series based algorithms, predicting microbe concentrations, interaction networks and stable states in perturbed and unperturbed populations. MDSINE is complementary to the work described here for several reasons: (i) MDSINE does not present a transition model between states; (ii) MDSINE results are not perturbation-focused, only considering a yes/no perturbation over time and (iii) MDSINE utilizes Maximum-Likelihood and Bayesian algorithms, compared to the Markovian algorithms employed in this work. Additional discussion regarding MDSINE is available in Supplementary Material. Martí *et al.* (2017) analysed variability in the microbiome, and also predicted microbiome transitions. Our work differs, however, in that: (i) we utilize a data-driven approach, versus their parametrized mathematical model; (ii) their work was focused exclusively on human gut, whereas our approach has a wider application range and (iii) the objectives of their work were to differentiate healthy and disease states, whereas the work presented here is focused on the effects of perturbations, and estimating the resulting state transitions. Finally, TIME (Baksi *et al.*, 2018) is a microbiome time series visualization tool that interactively allows biologists to show variations in terms of different metrics (such as diversity, abundance or stability). This, again, is distinct from the work presented here in that we aim to suggest consequential interventions, rather than simply observe temporal changes.

Markov Decision Processes (MDPs) have a history of use in medicine, for example, suggesting course-of-treatment within clinical decision support systems (Capan *et al.*, 2017; Sonnenberg and Beck, 1993). Here, we apply MDPs in a novel approach to the analysis of microbiome dynamics, and decision-support regarding directed interventions aimed at microbiome engineering.

## 2 System and methods

### 2.1 MDPbiome

**Fig. 1.**
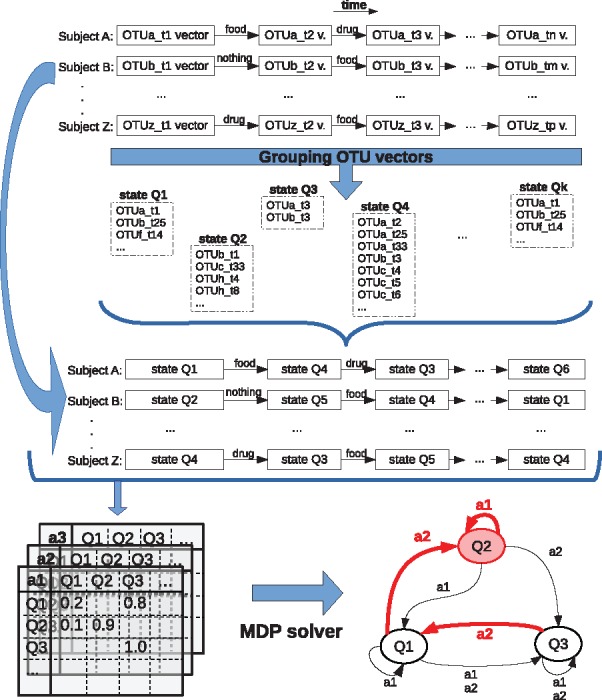
MDPbiome general schema for identifying a temporal sequence of microbiome states, influenced by perturbations, and how this may be employed to suggest a temporal sequence of actions aimed at achieving a defined goal

Figure 1 outlines our proposed solution, the MDPbiome system. We represent microbiome time series as a state transition diagram with actions, and solve the MDP to determine the optimal strategy of sequential interventions that will lead the microbiome to a goal state.

The input to MDPbiome are time series of microbiome data (see Fig. 1, top), where each series corresponds to a different subject (e.g. an individual patient). Each time point in the series is a microbiome sample, represented by an Operational Taxonomic Unit (OTU) vector. The number of time points and frequency of sampling may differ between subjects. Each transition between two time points is labelled with an external perturbation that occurred in that interval, such as some specific dietary intake, drug, probiotic treatment, etc. MDPbiome then presumes that this perturbation is causative of any composition change between those time points.

To create an MDP, we convert the time series of OTU vectors to a time series of microbiome states, preserving the transitions labelled with actions, and where each state is a group of microbiome samples. Next, from the time series of microbiome states, we obtain a transition probability table for each perturbation (Fig. 1, bottom left), and represent this as a transition diagram, labelling each transition with an action and its frequency. Finally, we apply an MDP solver to identify the optimal policy, i.e. a path to the goal state through the transition diagram (Fig. 1, bottom right).

We have implemented this workflow in a system called MDPbiome. In addition to the optimal policy, the system provides a set of quality control and visualization outputs, including evaluation of the policy reliability, and set of diagrams and graphs for visual analysis and interpretation of MDP elements and their related microbial data.

### 2.2 Markov decision process (MDP)

Markov Processes describe systems with stochastic transitions between discrete-time states. MDP extends Markov processes, including at each time step a decision point where an ‘agent’ can choose from a set of actions. Even though these actions might have stochastic effects, the model considers that these decisions affect the dynamics for transiting between states (Bellman, 1957; Howard, 1960; Puterman, 1994). In a basic Markov Process, the probability of the transitions between states are defined in a bi-dimensional square state-matrix. In an MDP, actions are an added dimension to the matrix. An MDP is formally defined as a tuple 〈S,A,T,R〉, where:

- S is a set of finite states
- A is a set of finite actions
- $T:S\times A\to P(S)=Pr(s_{t+1}|s_{t},a),\;\forall s\in S,\forall a\in A$
- R(S)→ℜ

T corresponds to the transition table that accounts for the probability of going from *s_t_* to *s_t_*_+1_ in the next time slot, given action *a* has been applied. R is the reward function, which represents how ‘good’ a state *s* is. A solution to an MDP is a policy (π:S→A), i.e. a mapping from states into actions that is used as the basis for decision making. The optimal policy $\pi^{*}$ for an MDP is the policy that maximizes the expected sum of rewards. The optimal policy is usually computed with a dynamic programming algorithm such as value iteration or policy iteration (Bellman, 1957; Howard, 1960).

### 2.3 MDP for longitudinal metagenomics

In our approach we represent longitudinal metagenomics with perturbations as a generic MDP=〈S,A,T,R〉. This involves adequately defining the four elements of the MDP, then solving it to obtain the optimal policy.

#### *2.3.1* Step 1: Pre-processing microbiome data

Metagenomics pre-processing steps are necessary, but not standardized, and differ between laboratories. In this work, pre-processing is applied to the OTU table following the methodology described in David *et al.* (2014), for longitudinal microbiomes. It involves removing OTUs not present in any samples (i.e. whose sum is 0), from other body sites, other donors, etc.; removing samples due to suspicion of experimental noise or contamination (as defined per experimental procedure); removing samples with low read-counts (<10 000); and normalizing the OTUs. For more details about pre-processing and normalization, see Supplementary Material or David *et al.* (2014).

#### *2.3.2* Step 2: Defining MDP states

The states considered by MDPbiome could be defined using any existing pre-definition of states (e.g. stages of treatment) or in the absence of an external definition, we utilize our own algorithm for robust clustering of OTU vectors based on the similarity of their microbial abundance composition (García-Jiménez and Wilkinson, 2018).

Though, clearly, microbial populations would evolve in a continuous manner, a discretization approach is a plausible simplification that makes modelling analysis viable. Many non-discrete processes (including biological ones) are studied with discrete computational and mathematical models (Faust and Raes, 2012; Faust *et al.*, 2015), and we do so here when defining states. It is important to note, however, that our defined MDP microbiome states are not synonymous with the concept of ‘enterotypes’, where the latter concept is limited to the gut microbiome, is highly controversial, and ill-defined (Costea *et al.*, 2018). Rather the states we describe here could appear within a single enterotype, and/or within any microbiome in any cavity or niche/environment. Gonze *et al.* (2017) proposed multi-stability as a reason for the existence of different states. Costea *et al.* (2018) concludes that ‘clustering can provide useful insights into some microbiome datasets, even when not strongly supported statistically’. Further supporting our definition of states in MDPbiome, Brooks *et al.* (2017) demonstrated there are no significant differences among distinct clustering schemas in terms of preserving the patterns of transitions in community state types in the vaginal microbiome; and Turroni *et al.* (2017) determined the existence of ‘shifting between different steady states’ in human gut microbiota dynamics of astronauts. See García-Jiménez and Wilkinson (2018) for additional discussion of discrete states in microbiomes.

#### *2.3.3* Step 3: Defining MDP actions

Having controllable perturbations as part of the model enables decision-making points to guide microbiome towards a desired goal state. The definition of what constitutes an ‘action’ (i.e. different values of a perturbation) depends on the particular experimental question. Briefly, actions can be binary, for instance, sexual practices (see Section 2.5.2); nominal, for example, salmonella vaccine and probiotic combined treatments in chicks (see Section 3.4); or numerical after discretizing, such as breast milk intake in infant gut (see Section 2.5.3). Since the set of actions in an MDP must be finite, we must discretize any continuous values in perturbations. We represent and solve a different MDP for each perturbation. Even though concurrent perturbations might not be independent in reality, this is a practical approximation, given that usually there is insufficient data to build an MDP that models combined actions. For few perturbations, we build set A as the cartesian product of the actions in the single perturbations. This alternative makes sense when there are a reasonable number of transitions for each combined action as discussed in Section 3.4.

#### *2.3.4* Step 4: Defining MDP transitions

We model the transition table T as a set of multinomial distributions that are estimated from the transitions observed in the available data. For each perturbation and subject, we split the metagenomics time series into triples $\left(s_{i},a_{j},s\prime_{k}\right)$ that corresponds to an observation where the microbiome was in *s_i_* and reached state $s\prime_{k}$ under the effects of action *a_j_.* Thus, probabilities of reaching a particular state is estimated as

$$P(s\prime_{k}|s_{i},a_{j})=\frac{counts(s_{i},a_{j},s\prime_{k})}{\sum_{k=1}^{\left|\text{S}\right|}counts(s_{i},a_{j},s\prime_{k})}$$

where the counts correspond to number of times the triples occur in the whole set of samples.

#### *2.3.5* Step 5: Defining MDP goal states

Our reward modelling only depends on states. MDPbiome defines two reward schemas to construct reward functions focused on either pursuing a desirable ‘goal’ state(s) or avoiding an undesirable ‘bad’ state(s). As input, the system needs a utility vector U(S) that establishes a numerical state preference. The schemas are defined as follows

RBest(s)=1, s=argmax(U(S))R¬Worst(s)=1, ∀s|s≠argmin(U(S))

having $R_{i}=0$ for any other case not holding the condition. $R_{Best}$ will guide the policy towards the best state, and $R_{\neg Worst}$ will guide decisions to avoid the worst state. Given that deciding the goal standard is not trivial for many domains, MDPbiome offers a default behaviour in which U(S) is set to alpha diversity (*α_div_*) average of the microbiome samples belonging to each state. In this case, for instance, $R_{Best}$ will lead to policies that pursue the highest diversity microbiome state.

#### *2.3.6* Step 6: Solving the microbiome MDP

Solving an MDP consists of finding an optimal policy $\pi^{*}$ that maximizes the cumulative expected reward for any given state. Here we apply the algorithm value iteration, a dynamic programming algorithm used to find policies for MDP with indefinite horizon. Our implementation uses the *MDPtoolbox* (Chadès *et al.*, 2014) R package as the base solver.

### 2.4 Evaluation technique

The state transition probabilities in our MDP models are estimated from the transition counts of the observed data. Thus, we consider it necessary to employ two evaluation procedures to measure how reliable the policies are. First we evaluate the stability of the resulting optimal policy given the uncertainty in the input data, specially under infrequent observed transitions. Then, in addition, we evaluate if the policies are general or particular amongst individuals.

#### *2.4.1* Sensitivity analysis on input data

We apply a sensitivity analysis on the estimated transition probabilities to measure the stability of the optimal policy, similar to (Chen *et al.*, 2017) but for an indefinite horizon for MDPs. The uncertainty of multinomial estimates is frequently modelled using the Dirichlet distribution with hyper-parameters that coincide with the observed transition counts (Tetreault *et al.*, 2007). Our evaluation procedure consists of generating 1000 transition tables ${\hat{T}}_{i}$ by sampling from the Dirichlet distributions corresponding to the observed transition counts. Each ${\hat{T}}_{i}$ is used to compute an optimal policy with value iteration. Finally we compute the ratio of actions that remain the same as in the original optimal policy. The higher the ratio, the more stable the optimal policy will be. We also compute the stability ratio of each individual action to identify if there are particular states in which one action should have a clear preference over the others to induce a microbiome state change.

#### *2.4.2* Policy generality amongst subjects

To make the most of the available data we perform a leave-*one*-out cross-validation (LOOCV). Given a dataset of *n* subjects, the procedure iterates *n* times, considering at each iteration *i*, all samples except those of subject *i.* Thus, each subject can be associated to an optimal policy $\pi_{i}^{*}$ computed with data not related to this subject. To measure the performance, we get from each subject time series, the set of transitions (s,s′) and then compute the number of transitions that lead to the same or better state (i.e. U(s)≥U(s′)) when the policy is applied in *s* and when is not. If the frequency of ‘good’ transitions following the policy is higher than when not following the policy we can infer that the policies are changing the *a priori* probability of these transitions and therefore they are general enough to be applied to out-of-sample individuals. As in the reward function, the *α_div_* of clusters is used as the utility function when an alternative sorting criteria is not provided.

### 2.5 Datasets

Few public longitudinal microbiome datasets include sufficiently frequent sampling and associated meta-data to allow perturbation studies. Table 1 summarizes the characteristics of each dataset for which our MDPbiome system could be applied, as the next subsections explain.

**Table 1.** Longitudinal microbiome datasets modelled as MDP

Ecosystem	Chick gut	Vaginal	Infant gut
References	Ballou (2016)	Gajer (2012)	LaRosa (2014)
No. samples	119	937	922
No. taxa	1583	298	29
No. subjects	24	32	58
Time points	6 in 4 weeks	2 × 16 weeks	Different
MDP.S	2	5	4
MDP.$A_{i}$	{salmonella, probiotics}	sexual practices {oral, toy,…}	{breast-milk, antibiotics}
MDP.$\left|T_{i}\right|$	94	905	893
U, MDP.R	Mature chick	Not bacterial vaginosis	Highest *α_div_*

#### *2.5.1* Chick gut microbiome

Ballou *et al.* (2016) studied the response of different treatments (Salmonella vaccine and/or probiotics) in the chick gut, during their first month of life. The data was downloaded from the Qiita repository (*http://qiita.ucsd.edu* study no.10291). The actions A are Salmonella vaccine (s) versus control (c); and probiotic supplement (0.1% Primalac) (p) versus (c). The control (c) is the lack of vaccine or probiotics, respectively. Salmonella vaccine was given at the outset of the study, prior to the day 0 sampling, and we consider the effect of the vaccine to remain throughout the time series. Probiotic was mixed with food on every day of the experiment. Thus, the action is the same for the same subject in its time series.

#### *2.5.2* Vaginal microbiome

Gajer *et al.* (2012) analyzed the dynamics of the vaginal microbiome taking samples from 32 women over several weeks. The OTU table and the clusters come from their supplementary Table S2 (Gajer *et al.*, 2012). The data counts were pre-processed, and normalized to a sum of 100 per sample, thus representing relative abundances. As such, we did not apply our pre-processing step to this dataset.

The actions set A was composed by the available meta-data related to the hygienic and sex activities that could perturb the vaginal microbiome ($A_{i}$* = {Yes, No}* x {anal sex, digital penetration, douching, lubricant, oral sex, sex toy, tampon, vaginal intercourse}). As MDP states, we take community state types defined by the original study authors (S* = {I, II, III, IV-A, IV-B}*). The IV-B group concentrates most of the high and intermediate Nugent categories indicating the greatest risk for the Bacterial Vaginosis disease (Nugent *et al.*, 1991). The preferred reward schema in this scenario is $R_{\neg Worst}$, focused on avoiding a non-desired state related to bacterial vaginosis. Therefore, we take the inverse of the average Nugent score per state as utility function U(S).

#### *2.5.3* Infant gut microbiome

The La Rosa *et al.* (2014) dataset includes 58 pre-term babies who were stool-sampled at variable time points until a month and a half. The meta-data provides us with two different perturbations: amount of breast milk volume and antibiotic use. The first one is provided as 4 discretized values (*0%, low: <10%, med: 10*–*50%, high: >50%* of enteral volume). The samples for 0% of volume represent <2% of the total samples, thus we merged this category with *low: <10%.* The second perturbation corresponds to antibiotics administration in the last 3 days (yes, no). We used *α_div_* as the utility function and evaluate both reward schemas.

## 3 Results

The following sections describe the most informative results of the application of our MDPbiome system to the three datasets. The complete set of MDPbiome outputs (text and figures) for all datasets and all configurations are available at *https://tomdelarosa.shinyapps.io/mdpbiome/*.

### 3.1 General results

#### *3.1.1* Chick gut microbiome

Ballou *et al.* (2016) indicated there were no differences in the health of the chicks at the end of the study. Therefore, lacking additional domain knowledge, we defined the MDP states following our robust clustering procedure (García-Jiménez and Wilkinson, 2018). From this, we observed that the states primarily mirror the chicks’ age (i.e. sampling day): there is one state (called *birth*) with samples from chicks in their first days of life (mainly, day 0–3), and another state (*mature*) with microbiomes of all the chicks aged 2 or more weeks (mainly day 14 and 28). This split according to age is in agreement with the Ballou *et al.* analysis, where samples do not differ by any other criteria, including the experimental conditions (vaccine/probiotics). Both states contains samples from all treatments. The mature state is much more diverse than the birth state with respect to microbial composition (mean *α_div_*: 3.29 ≫ 1.40). Moreover, setting the highest diversity as the goal brings our chick MDPbiome model into agreement with child microbiome evolution studies (Dominguez-Bello *et al.*, 2011; Oakley *et al.*, 2014), which begin with an empty or very low-diversity microbiome (such as our *birth* state), while achieving maximum diversity in adulthood (our *mature* state).

**Fig. 2.**
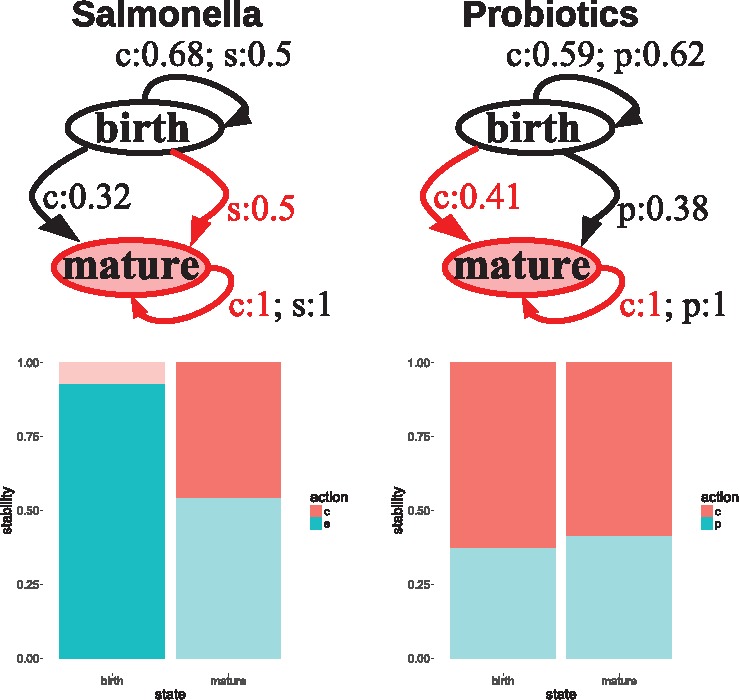
MDP diagrams with optimal policy and barplots with stability ratio per state and perturbation to chick gut microbiomes. In the diagram, red arrows represent the MDP solution; and the goal state is that highlighted in red. In the barplots, strong colors represent the optimal policy and faint colors the non-optimal ones. The *s(p)/c* actions mean salmonella vaccine(probiotics) yes/no (c, control), respectively

Figure 2 shows how to engineer the chick gut microbiome with salmonella vaccine (left) and probiotics (right) as perturbations. In the case of salmonella, the recommendation is to administer the vaccine to reach the mature state, with a very high stability (see dark blue bar in Fig. 2, left). When the perturbation is probiotics, the highest probability to reach the goal state is ‘c’ (no probiotics) (see 0.41 > 0.38 in diagram) also with a stability higher than the alternative action of ‘p’ (red bar larger than the blue one). In the mature state, with both perturbations, all actions have the same probability (1) to maintain that state, because chick microbiomes do not return to a less diverse state post-maturation.

In conclusion, the preferred policy is to administer salmonella vaccine to accelerate maturation; and not to feed with probiotics (although the policy regarding probiotics is less conclusive).

#### *3.1.2* Vaginal microbiome

This scenario differs from the other datasets in that most states are considered ‘healthy’, thus the preferred goal is avoidance of a disease state. As Section 2.5 describes, state IV-B was identified as the non-desired state, and the MDP was designed to provide a policy in which the path is the (series of) action(s) that minimize the risk of reaching it.

As a general overview, all states except IV-A have >85% likelihood of remaining in the same state, regardless of action. Therefore, the most common model-behaviour is to maintain the same state, regardless of perturbation and state-to-state transitions are less frequent. MDPbiome transition diagrams (see *shinyapp* online) show a high degree of maintenance of the disease state IV-B, suggesting that is clearly difficult to escape from it without specific medical intervention.

**Fig. 3.**
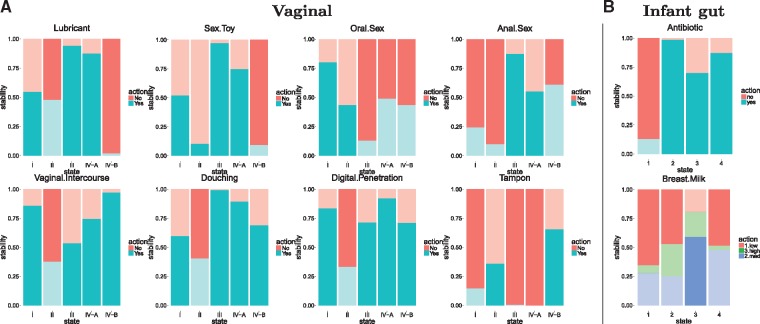
MDPbiome recommendations to reach the defined goal, in terms of stability ratio of the optimal policy of single perturbations given each particular state. (**A**) Goal = avoid bacterial vaginosis. (**B**) Goal = highest *α_div_.* (A and B) Each independent graph represents an external perturbation that could influence the vaginal or infant gut microbiome, respectively. Columns represent the microbiome states. Stronger colors represent the optimal policy and faint colors the non-optimal ones. Red bars correspond to ‘no’ and blue ones to ‘yes’, with the exception of breast milk which is discretized into three categories

Figure 3A shows the optimal policy and its stability per state (each of the five columns represents a state) and perturbation (eight graphs, one for each perturbation) of the vaginal microbiome. Here, we focus our analysis on the last column of each plot, corresponding to the state to avoid with $R_{\neg Worst}$. The top row collects the perturbations where the optimal policy is ‘perturbation-no’, in decreasing order, so lubricant and sex toy are not recommended to recover from bacterial vaginosis, with high stability. However, anal sex-no does not evince enough stability to be considered a strong policy to follow. These results are in agreement with Brotman *et al.* (2010), who identified several ‘risk factors’ for bacterial vaginosis, including the use of a lubricant and rectal sex.

#### *3.1.3* Infant gut microbiome

In the absence of domain knowledge, we again defined the MDP states applying our robust clustering procedure. Characterizing the four identified states, all have a very low *α_div_* (<1), which is consistent with the newborn origin, where the gut microbiome is beginning to be populated. This diversity measure is the only available information to define the goal state as being the highest *α_div_* state in $R_{Best}$ rewards (state no.1, *α* = 0.85) and the lowest one in $R_{\neg Worst}$ (state no.4, *α* = 0.35).

State 1 is mainly composed by Clostridia, state 2 by Gammaproteobacteria, 3 by Bacilli and 4 by Bacteroidia. According to La Rosa *et al.* (2014), each subject should follow the sequence Bacilli (3) → Gammaproteobacteria (2) → Clostridia (1). State 4 could correspond to outliers, because it only includes samples from two infants taking >50*%* of breast milk.

The most relevant conclusions in the infant gut microbiome are that, to preserve the most diverse state and the final one in the maturation sequence (state 1), *no* antibiotics should be administered, with the stability of this recommendation higher than 90% (see large red bar in Fig. 3B, top). However, regarding milk intake the stabilities are less conclusive. These observations are in-agreement with studies indicating that antibiotics cause much more disturbance in microbiomes than dietary interventions (Costea *et al.*, 2018).

### 3.2 Evaluating policy stability

Table 2 shows the stability ratio (see Section 2.4.1) for the optimal policies per different dataset, perturbation and reward schema. This ratio computes, in terms of actions, the similarity that the policies obtained with transition tables from the Dirichlet sampling would have with the optimal policy computed with the original probability estimates. This aggregated value can be seen as a measure of the reliability of the MDPbiome policy. In terms of microbiome engineering, it means how invariant the recommendation for applying a particular perturbation would be. Although there are differences between datasets, perturbations and rewards, most values are in a 60–85% range, showing MDPbiome suggestions achieve high stability along multiple distinct cases. The minimum threshold of stability depends on the number of values of each perturbation ($\frac{1}{\left|\text{A}\right|}$), with 50% for yes/no perturbations and lower for perturbations with >2 values, such as the combined treatments in infant gut microbiome, where even 26% stability is greater than choosing a random policy ($\frac{1}{6}$). In the chick gut dataset, $R_{B}$ is equivalent to $R_{\neg W}$ because there are only two states. The differences in this case are due to sampling and therefore negligible.

**Table 2.** Aggregated optimal policy stability ratio for perturbations

**Chick gut**	
	$R_{B}$	$R_{\neg W}$
Salmonella	0.68	0.78
Probiotic	0.59	0.60
Combined	0.48	0.43
**Infant gut**	
	$R_{B}$	$R_{\neg W}$
Breast milk	0.56	0.59
Antibiotics	0.84	0.87
Combined	0.54	0.26
**Vaginal**	
	$R_{B}$	$R_{\neg W}$
Anal sex	0.58	0.70
Digital penet.	0.77	0.79
Douching	0.65	0.75
Lubricant	0.76	0.76
Oral sex	0.75	0.64
Sex toy	0.85	0.66
Tampon	0.87	0.77
V. intercourse	0.70	0.76

A lower stability ratio often means there are too few samples for the number of distinct actions of a perturbation, and therefore the transition probability table is not reliable because of the uncertainty from the input data. In other cases, the stability ratio is affected when transitions resulting from different actions have a similar probability and associated reward. Here, small changes in the input time series could produce a change in the recommended strategy. For this reason, we consider the interpretation of the per-state stability of actions relevant, as indicated by the stability barplots (such as Figs 2 and 3).

### 3.3 Evaluating policy universality

The datasets used in our study were not originally designed for guided perturbation analyses, and had terminated prior to the initiation of our study. Therefore, there is no direct way to test policy accuracy by imposing the guidance indicated in the optimal policy on a test subject. Thus, our approximation of this ideal test procedure is, with existing data, to relate the compliance the subject has in following the policy, with the number of times the subject moves to a better, equal or worse state, compared to when the subject does not follow the policy.

**Fig. 4.**
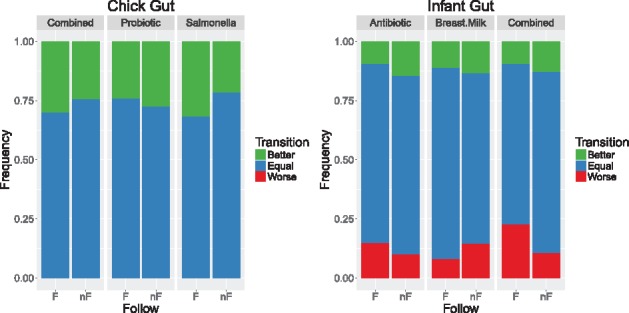
Frequency of categorized transitions when following, or not, the optimal policy. F: following the MDPbiome policy, nF: not following it. Better, equal and worse state-transition is defined considering *α_div_* for sorting states

Figure 4 shows the results for chick and infant gut datasets, and Supplementary Figure S1 for vaginal microbiome. The LOOCV results provides confirmation that the infant gut microbiome is quite stable regardless of perturbations (75.5%). Nevertheless, there is some evidence to support that the policies are generalizable across individuals (see Fig. 4, right): 91.9% of the transitions for breast milk intake result in the same or in a better state (blue + green of central columns) when following the policy (F), versus 85.5% when the policy was not followed (nF).

In the chick guts, there is no case where the microbiome gets worse (no red bar), because there are only two states and all chicks move from birth to a mature state (goal) eventually. When the perturbation is salmonella vaccine or combined, following the policy reported better results than not following it (see larger green bars in F than nF in 1st and 3rd pair of columns in Fig. 4, left); however, when the probiotic is the single perturbation, our generality evaluation indicates that not following the policy is better.

### 3.4 Combining actions

In the chick dataset, we could merge simultaneous perturbations because the data corresponds to an exhaustive collection of all possible combinations of states and perturbations. Thus, actions go from salmonella {yes, no} and probiotics {yes, no} to one combined perturbation equivalent to the treatment with four different values {cc, cp, sc, sp}.

**Fig. 5.**
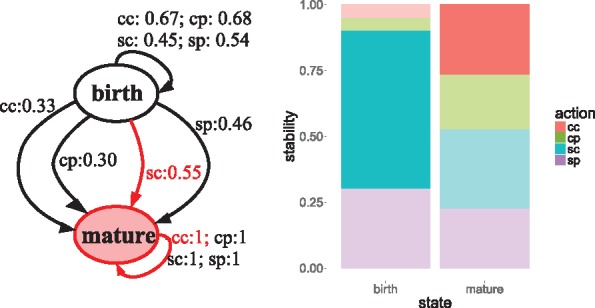
MDP diagram with optimal policy and barplot with stability ratio per state and combined perturbation to chick gut microbiomes. Same legend than in Figure 2

When we combine perturbations to define the MDP actions, the aggregated stability ratio is lower than considering the perturbations independently (see Table 2); due to the reduction in the completeness of the transition table; the same transitions must fulfill a table with higher dimensions. This demonstrates that combining simultaneous perturbations in an unique MDP could be viable if there are sufficient data about all combinations of perturbations, however it could entail lower stability in the optimal policy. The MDP diagram in Figure 5 points out that ‘sc’ is the action with the highest probability to reach the mature state. The barplot in Figure 5 shows that the optimal policy in state *mature*, i.e. ‘cc’, is not the one with the highest stability, because regardless of the action, there is the same probability to preserve the mature state. While in the birth state, the optimal policy, and the most stable, are clearly the same: to administer salmonella vaccine, without probiotics. Therefore, we could conclude that an adult microbiome, with more diversity, is reached earlier with the salmonella vaccine treatment without probiotics.

In the infant gut dataset, the two perturbations (milk and antibiotics) could also be represented as a combined perturbation with six actions to study the effect of simultaneous perturbations. In this scenario, there are many actions that split the available transitions, resulting in very few data points per action, leading to a decrease in the stability and generality of the optimal policy when a combined perturbation is considered versus the individual ones (see last column Table 2 and Fig. 4).

In the vaginal microbiome it was not feasible to combine perturbations because the number of combinations (2^8^) is too high, resulting in many state transitions not being observed in the dataset.

### 3.5 Re-defining states

We examined the consequences of changing the MDP state definition for the same dataset. We filtered the subset of taxa (dominant, non-dominant) used by our robust clustering algorithm. We defined dominant taxa as a percentage (1% or 0.5%) of the most frequent taxa; and the complementary taxa are the non-dominant subset.

**Fig. 6.**
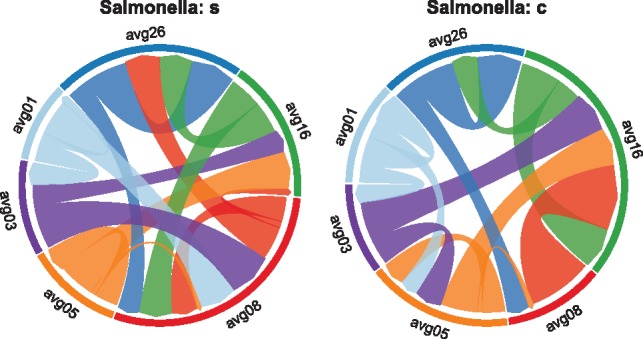
Chick gut microbiome state transitions when clustering non-dominant taxa at 1%, with the Salmonella vaccine perturbation. The ribbon-arrow points out the directionality of the transitions, and ribbon width represents the frequency of transition. Left circle shows the transition probabilities when the perturbation is applied, and right one when it is not. The colors represent each state, labelled with the average chick age of the samples within

In the Ballou *et al.* (2016) dataset, when all taxa are clustered, the result was two states (young and mature chicks). However, considering only non-dominant taxa at 1%, six clusters are robustly identified, grouping the chicks based on similar age. In fact, the *α_div_* of these states change in parallel, increasing with the average age of the chicks in each cluster. In both clusterings (all and non-dominant 1%), a consistent MDPbiome optimal policy is preserved—to administer salmonella vaccine to reach maturation as quickly as possible—despite the total number of states being tripled. Analyzing transition probabilities in the non-dominant taxa at 1% clusters, with salmonella-yes (Fig. 6, left), we observe transitions between non-consecutive states (see more crossing lines in the middle of left than right circle plot, such as from avg1 to avg8, avg3 to avg8 and avg8 to avg26) while with no vaccine (‘c’) the transitions are mainly sequential, meaning a slower maturation. Thus, this provides a biological explanation for how salmonella vaccine accelerate maturation that was not (and could not be) detected in the original study. In chick microbiome, it seems that the use of non-dominant species for clustering presents a higher-resolution separation of microbiome states, that nonetheless behaved in the same manner as the more coarse analysis using all taxa.

Nevertheless, besides useful information such as that just described, no conclusive determination could be made regarding the MPDbiome policy stability and generality when filtering data along the lines of dominant/non-dominant taxa. Comprehensive analysis of additional, larger, purpose-designed datasets will be required to derive more general conclusions.

## 4 Discussion

The MDPbiome approach has a variety of positive features. First, it can be applied to a wide diversity of datasets and external perturbations. Second, MDPbiome can evaluate multiple (>2) microbiome states. As such, the more common ‘healthy’ vs ‘unhealthy’ categories in typical microbiome variability studies (Martí *et al.*, 2017) could be examined with greater granularity, with the ability to identify, for example, healthy sub-states that are nevertheless more at-risk of transitioning into an unhealthy one (e.g. state IV-A in the vaginal microbiome analysis). Third, MDPbiome allows a variety of different questions to be asked of the same dataset. For example, the MDP may be optimized to reward microbial diversity (i.e. the default behaviour), or may alternatively be set to optimize for recovery from a disease.

Stability evaluation of the MDPbiome policies indicates the degree of confidence that can be attributed to these policies. Thus, we can recognize whether the number of subjects and/or variability of transitions is sufficient to cover all possible paths in the MDP diagram, and therefore derive stronger conclusions. Similarly, generality evaluation measures the predicted performance of the policies when prescribed to subjects not included in the original study.

MDPbiome results may also be used for hypothesis-generation, for example, predicting interaction between the specific microbes present in two states. For example, if a transition between state *Q1* and *Q2* is defined with high probability and stability for a given perturbation *x*, it could mean that *x* might facilitate the increase of the predominant bacteria in state *Q2* or be a detriment to the predominant bacteria in state *Q1.* Moreover, state-transitions in the absence of perturbation (action-negative) may reveal patterns of competition or cooperation between the species in each state.

Selection of an appropriate MDP representation of a microbiome entails careful consideration, in particular because the Markov property requires that the next state depends only on the current state and action, regardless of previous states or actions. In our temporal microbiome scenario, this might not be completely true since interventions, such as food or drug intake, may have prolonged effects that last over many observational cycles. Nevertheless, for this study we consider this simplification to be acceptable, and that in general the predictive power gained by the MDP principles is worth this potential limit or noise in its sensitivity.

As with most population-based studies, the quality of the transition diagrams are highly dependent on the quality and quantity of the available data. With even a few 〈transition, action〉 pairs we can generate a meaningful estimation of the transition probability. However, in some cases these 〈transition, action〉 observations are insufficient. This prevents us from deriving even a basic MDP model, for instance in the dataset from David *et al.* (2014), where there is a lack of annotated transitions between different states.

MDPbiome obtains a policy independently for each perturbation, and for combined perturbations only when there are (i) few individual perturbations and (ii) sufficient transitions to estimate the probabilities. MDPbiome is, therefore, similar to other population-based studies, being limited by the trade-off of potential information gain from non-independent perturbations and the decrease in reliability due to sparse transitions for those combinations. Because we are not able to examine combinatorial perturbations in many cases, we are unable to assert that the application of a single external perturbation is sufficient to achieve a given state-transition; effectively, we cannot guarantee that perturbations are independent, neither in their effect, nor even in their application (i.e. two measured perturbations may usually or always occur simultaneously). Similarly, we cannot be sure that the different external perturbations that compose a combined-policy need to be applied together to achieve (or prevent) the state-transition.

Despite those drawbacks, there is a clear and pressing need to predict the effects of interventions on microbiomes, both within medicine and for industrial and environmental scenarios. This paper provides, to our knowledge, the first example of a MDP being used to explain how a microbial community will respond to any given intervention. We believe this provides the basis for a wide range of directed research, in particular with respect to microbiome-engineering for health in the medical domain.

Adding further flexibility in modelling will make MDPbiome a tool of interest for a wider scope of metagenomic studies. Alternative definitions of the MDP will better enable specific scenarios within future microbiome-engineering initiatives. For example, the reward function could be defined as R:S×A→ℜ (Puterman, 1994), to indicate that there is a preference on reaching a state using a particular action. MDPs could be also modelled in terms of cost rather than rewards, or with cost and rewards at the same time. A cost function C(S,A), could be used to represent the economic cost of a treatment that tries to recover a healthy microbial state. Further, a finite horizon MDP is a practical approach for finding a n-steps policy in which a net monetary benefit can be computed, where each applied perturbation entails a high cost (e.g. expensive drugs).

## 5 Conclusions and further work

Costea *et al.* (2018) imagined a computational tool for suggesting perturbations to modulate the microbiome to move from a disease to healthy state, but noted that no such tool existed. MDPbiome builds a model that suggests a ‘prescription’ of external perturbations that should be applied to a given microbiome that will result in its navigation through a subset of healthy or acceptable states, avoiding disease or other undesirable states, finally reaching a goal state. This manuscript confirms that, given sufficient data, MDPbiome can be applied to a diversity of temporal metagenomics datasets, such as the three distinct domains whose solution in terms of microbiome dynamics was found using the proposed MDP strategy, and to a wide range of perturbations types.

The main knowledge contributions of this manuscript are: (i) the inclusion of actions in microbiome state transition diagrams; (ii) prediction of the sequence of external perturbations required to preserve or to reach a desired microbiome state or to avoid an undesirable one; (iii) techniques to evaluate the stability and generality of the recommendation policies given a new dataset; (iv) demonstration of our algorithms flexibility in definition/selection of the goal, for example, being desirable or undesirable, or even dependent on a continuous utility function defining the reward; (v) demonstration that, given sufficient data, combinatorial perturbations can be evaluated. The primary technical contributions of this manuscript are: (i) R software that can be easily configured to apply to a new dataset, (ii) a set of visualizations to facilitate further third-party analysis of the multiple outputs of MDPbiome applied to these three datasets.

The power of MDPbiome will improve as increasingly rich microbiome samples become available in the next years. Nevertheless, MDPbiome may already be used to generate novel hypotheses for research into temporal microbiome dynamics under perturbations. Future work will enable MDPbiome to include weights in the MDP, allowing it to represent, for example, the cost of a specific policy/perturbation, thus providing not only optimal but also cost-efficient policies. Other improvements will involve substituting OTUs by a higher resolution measures (sub-OTU) recently defined by amplicon sequence variant (ASV) (Callahan *et al.*, 2017). State-definitions could be extended from phenotypic to molecular, using transcriptomics, metabolomics, or other high-throughput biomarkers, and the range of applicability will be expanded into the industrial and agricultural domains. Finally, we will explore the utility of MDPbiome in the examination of multi-population studies, for example, to explore the influence of the gut microbiome on the microbiomes in other body cavities.

## Supplementary Material

Supplementary Data
