# Supplementary material for: MDPbiome: microbiome engineering through prescriptive perturbations
Source: Bioinformatics. 2018 Sep 8;34(17):i838–47. doi: 10.1093/bioinformatics/bty562 (PMC6129268; doi:10.1093/bioinformatics/bty562)
Supplement: Supplementary Data [file bty562_supplementary_material.pdf]

Supplementary Material for: MDPbiome: microbiome engineering through prescriptive perturbations

Beatriz García-Jiménez, Tomás de la Rosa and Mark D. Wilkinson

April 11, 2018

Methods

Pre-processing and normalizing microbiome data

Pre-processing is dependent on the specific methodologies/technologies for sample preparation and data generation, and cannot be prescribed in a generic manner for all datasets. This is particularly relevant for this study, since we utilize datasets generated by other laboratories, each with its own intrinsic pre-processing requirements.

One of the most influential pre-processing steps is normalization of the OTU counts. We have selected *David et.al.*'s normalization (David, 2014), because it includes a log transformation which is recommended to preserve the relative microbe relations, taking into account the compositional nature of the data (Aitchison, 1982). We use the python code published by the authors (David, 2014) but we add a pseudo-count with "a value smaller than the minimum abundance value prior to transformation"(Costea *et al.*, 2014). In particular, the selected pseudo-count is one order of magnitude (base 10) less than the minimum abundance value, as recommended by Costea *et al.* (2014). For example, for a minimum abundance value of 1.623e-06, the pseudo-count will be 1e-07.

Markov Decision Process additional element

Sometimes, an additional element is also included in the MDP definition: $\gamma \in [0, 1[$; called the discount factor, which is a constant typically close to 1, used to indicate how important are future rewards compared to rewards obtained in the current state. We set a discount factor $\gamma = 0.9$ for future rewards, a typical value for this setting.

Datasets

Vaginal microbiome (Gajer *et al.*, 2012): additional details

Gajer *et al.* obtained the clusters in the vaginal microbiome dataset by hierarchical clustering, with Jensen-Shannon distance, with Ward linkage, cutting the dendogram with a k between 2 and 10, with the maximum silhouette inside this range. The maximum silhouette was at k=5, and thus they obtained 5 states.

The actions were collected by a curated visual inspection of the individual profiles of the dynamics of vaginal bacterial communities, from the 32 D-panels (one profile per woman) of supplementary material in Figure S5 (Gajer *et al.*, 2012) available on-line. We associated the external perturbation to the next sample taken, or the same day if it coincides, and this is then considered the 'action' between the two samples.

Although Brotman *et al.* (2014) used continuous-time Markov models to examine the same dataset (Gajer *et al.*, 2012), their approach differs in the type of Markov model (not an MDP), in the prediction goal (infection with human papillomavirus rather than bacterial vaginosis) and chiefly in that they did not correlate actions/perturbations with state-transitions.

Results

Evaluating policy universality in vaginal microbiome

Figure S1 shows the results of evaluating the generality of the MDPbiome policies in the vaginal microbiome dataset for the different perturbations. Figure S1 indicates that lubricant and sex toy policies are very general, with a large difference in going to equal or better states through following the policy versus not following it (see bottom of 4th and 7th pairs of columns, with shorter (F) and longer (nF) red bars). With respect to anal sex, oral sex and tampon-use, following the policy is better, while with digital penetration or vaginal intercourse the policy seems to not be universal among different subjects. In conclusion, the generality level of the policy depends on the microbiome dataset and the perturbation.

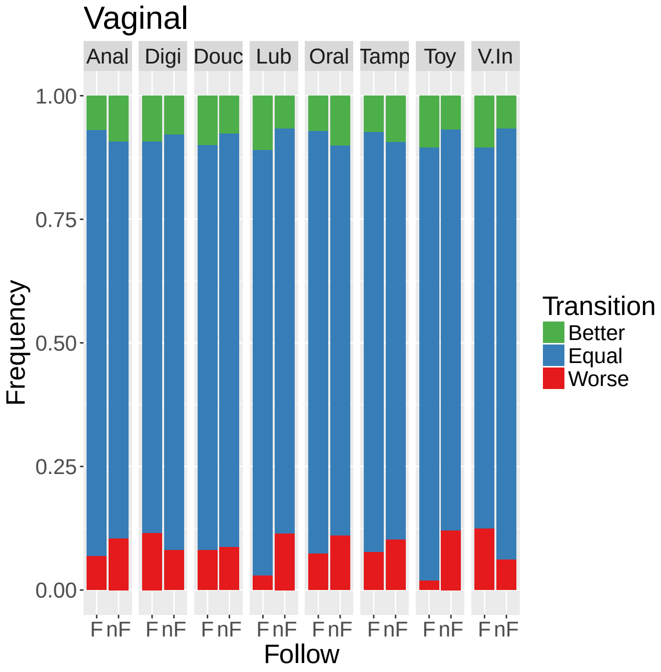

Figure S1: **Frequency of categorized transitions when following or not the optimal policy, in vaginal microbiome.** F: following the MDPbiome policy, nF: not following it. Better, equal and worse state-transition is defined considering inverse of the average Nugent score for sorting states.

Additional comparison with MDSINE

Here we define additional differences between MDPbiome and MDSINE, apart from those ones described in the main text. MDSINE requires qPCR and 16S data input, while MDPbiome only require the latter. MDSINE uses a generalized Lotka-Volterra model (gLV), where a transformation is applied (they called 'grading matching') to simplified the ordinal differential equation problem to a linear problem. Thus, given MDSINE gLV model, they developed additional algorithms to do predictions and evaluate their model. Similarly, given MDPbiome MDP model we obtain predictions (optimal policy) applying a MDP solver to our model, and we also develop: 1) our Dirichlet-based algorithm to evaluate our system, resulting in the definition of our optimal policy stability rate metric, individual per action and state, and in an aggregated way; and 2) a leave-*one*-out cross validation based algorithm resulting in our generality metric, that measures the frequency of following optimal policy compared to reach an equal, better or worse state. Regarding perturbations, MDPbiome suggests the perturbation to apply in a given state, while MDSINE detects if a perturbation (or interaction) occurred, comparing models with and without perturbations, or if the perturbation has effect or not. In conclusion, we could not quantitative comparing both systems because their assumptions, model input and outputs, resulting in different and not-comparable metrics.

References

Aitchison, J. (1982). The Statistical Analysis of Compositional Data. *Journal of the Royal Statistical Society. Series B (Methodological)*, **44**(2), 139–177.

Brotman, R. M., Shardell, M. D., Gajer, P., Tracy, J. K., Zenilman, J. M., Ravel, J., and Gravitt, P. E. (2014). Interplay between the temporal dynamics of the vaginal microbiota and human papillomavirus detection. *The Journal of infectious diseases*, **210**(11), 1723–1733.

Costea, P. I., Zeller, G., Sunagawa, S., and Bork, P. (2014). A fair comparison. *Nat Meth*, **11**(4), 359.

David, L. A. (2014). Normalizing microbiota time-series data. http://nbviewer.ipython.org/github/ladavid/mit_timeseries/blob/master/NormalizeDemo.ipynb.

Gajer, P., Brotman, R. M., Bai, G., Sakamoto, J., Schütte, U. M. E., Zhong, X., Koenig, S. S. K., Fu, L., Ma, Z. S., Zhou, X., Abdo, Z., Forney, L. J., and Ravel, J. (2012). Temporal dynamics of the human vaginal microbiota. *Science translational medicine*, **4**(132), 132ra52.
